# Quantitative Proteomic Analysis of the Response to Zinc, Magnesium, and Calcium Deficiency in Specific Cell Types of *Arabidopsis* Roots

**DOI:** 10.3390/proteomes4010001

**Published:** 2016-01-12

**Authors:** Yoichiro Fukao, Mami Kobayashi, Sajad Majeed Zargar, Rie Kurata, Risa Fukui, Izumi C. Mori, Yoshiyuki Ogata

**Affiliations:** 1 Department of Bioinformatics, Ritsumeikan University, Kusatsu, Shiga 525-8577, Japan; 2 Graduate School of Biological Sciences, Nara Institute of Science and Technology, Ikoma 630-0192, Japan; Mami_Kobayashi@skal.suntory.co.jp (M.K.); smzargar@gmail.com (S.M.Z.); kurata@bs.naist.jp (R.K.); 3 Centre for Plant Biotechnology, Division of Biotechnology, Sher-e-Kashmir University of Agricultural Sciences and Technology of Kashmir, Shalimar, Srinagar, Jammu and Kashmir 190025, India; 4 Division of Applied Life Sciences, Graduate School of Life and Environmental Sciences, Osaka Prefecture University, Sakai, Osaka 599-8531, Japan; su203024@edu.osakafu-u.ac.jp (R.F.); ogata@plant.osakafu-u.ac.jp (Y.O.); 5 Institute of Plant Science and Resources, Okayama University, Kurashiki, Okayama 710-0046, Japan; imori@okayama-u.ac.jp

**Keywords:** FACS, iTRAQ, OFFGEL electrophoresis, root specific cell types, 14-3-3 protein family, *Arabidopsis thaliana*

## Abstract

The proteome profiles of specific cell types have recently been investigated using techniques such as fluorescence activated cell sorting and laser capture microdissection. However, quantitative proteomic analysis of specific cell types has not yet been performed. In this study, to investigate the response of the proteome to zinc, magnesium, and calcium deficiency in specific cell types of *Arabidopsis thaliana* roots, we performed isobaric tags for relative and absolute quantification (iTRAQ)-based quantitative proteomics using GFP-expressing protoplasts collected by fluorescence-activated cell sorting. Protoplasts were collected from the pGL2-GFPer and pMGP-GFPer marker lines for epidermis or inner cell lines (pericycle, endodermis, and cortex), respectively. To increase the number of proteins identified, iTRAQ-labeled peptides were separated into 24 fractions by OFFGFEL electrophoresis prior to high-performance liquid chromatography coupled with mass spectrometry analysis. Overall, 1039 and 737 proteins were identified and quantified in the epidermal and inner cell lines, respectively. Interestingly, the expression of many proteins was decreased in the epidermis by mineral deficiency, although a weaker effect was observed in inner cell lines such as the pericycle, endodermis, and cortex. Here, we report for the first time the quantitative proteomics of specific cell types in *Arabidopsis* roots.

## 1. Introduction

The proteomic profiles of specific cell types have been intensively investigated in animal systems [1,2,3,4,5]. On the other hand, few studies have been conducted on plants [6,7,8,9]. Recently, fluorescence activated cell sorting (FACS) has been applied to proteomic analyses, to obtain proteome maps of particular cell types [8]. However, these studies have been limited because low-abundance proteins are difficult to identify, because of masking by peptides derived from abundant proteins when using high performance liquid chromatography coupled with mass spectrometry (LC-MS) analysis. To resolve this issue, we recently developed a method that combines FACS and OFFGEL electrophoresis [9]. OFFGEL electrophoresis allowed identification of a larger number of proteins by separating the peptides into 24 fractions prior to LC-MS. Furthermore, FACS is useful not only for collecting specific cell types, but also for decreasing protein populations. Therefore, the FACS-OFFGEL method is highly effective for identifying a large number of proteins, including those expressed in low abundance.

In the past decade, various methods of quantitative proteomics such as ICAT (isotope-coded affinity tag [10]), iTRAQ [11,12], SILAC (stable isotope labeling by amino acids in cell culture [13,14,15]), and emPAI (exponentially modified protein abundance index [16]) have been adopted in the field of plant science. These methods have been utilized to reveal several biological interests such as subcellular proteomes, protein–protein interactions, plant and organ development, environmental stimulus, and post-translational modifications [17,18]. Recently, we have established the combinational method using iTRAQ and OFFGEL electrophoresis to obtain quantitative information including low abundant proteins [19]. In this study, to elucidate the response of the proteome to zinc (Zn), magnesium (Mg), and calcium (Ca) deficiency in epidermal or inner cell lines, such as pericycle, endodermis, and cortex cells, we have further applied the FACS-OFFGEL and the iTRAQ-OFFGEL methods. Here, we report for the first time a quantitative proteomic analysis of a specific cell type in *Arabidopsis* roots. Using FACS, we collected protoplasts from *Arabidopsis* transgenic lines in which GFP was specifically expressed in epidermis (pGL2-GFPer [20]) or in inner cell lines (pericycle, endodermis, and cortex) (pMGP-GFPer [21]). The sorted GFP-expressing protoplasts were analyzed using the iTRAQ-OFFGEL method (hereafter, FACS-iTRAQ-OFFGEL). A total of 1039 and 737 proteins were identified and quantified in the epidermal and inner cell lines, respectively, that responded to Zn, Mg, and Ca deficiency. A remarkable number of proteins were found to be downregulated in epidermal cells by mineral deficiencies, suggesting that the epidermis is strongly affected by mineral deficiencies. However, the proteome of inner cells was not affected by mineral deficiencies, and appears to sustain normal cellular processes.

## 2. Experimental Section

### 2.1. Plant Growth Condition and Preparation of Protoplasts

Col-0, pGL2-GFPer and pMGP-GFPer seeds were sterilized and germinated on basal medium, 0-Zn, 0-Mg, or 0.5-Ca medium (see below for the detailed composition of each medium). These media contain 1.0% (*w*/*v*) sucrose and 1.2% purified agar (Nacalai Tesque, Kyoto, Japan). Approximately 1500 Col-0, 15000 pGL2-GFPer, or 15000 pMGP-GFPer seedlings were grown vertically for five days at 22 °C under 16-h light/ 8-h dark conditions. Protoplasts were prepared from roots as described previously [9]. Then, GFP-positive protoplasts (~5.0 × 10^6^) from pGL2-GFPer and pMGP-GFPer grown on each growth condition were sorted using the FACSAria III cell-sorting machine (BD). Protoplasts from Col-0 were used as negative control. The sorted protoplasts were collected by centrifugation at 500× *g* at 4 °C for 10 min, and then pellets were dissolved in 30 µL of iTRAQ buffer (Applied Science, Foster City, CA, USA).

### 2.2. Composition of Basal, 0-Zn, 0-Mg, or 0.5-Ca Medium

Basal or 0-Zn medium compose of 3 mM KNO_3_, 2 mM Ca(NO_3_)_2_•4H_2_O, 1.75 mM NaH_2_PO_4_•2H_2_O, 1.75 mM Na_2_HPO_4_•12H_2_O, 1.5 mM MgSO_4_•7H_2_O, 1.5 mM K_2_SO_4_, 67 µM Na_2_-EDTA•2H_2_O, 30 µM H_3_BO_3_, 10.3 µM MnSO_4_•5H_2_O, 8.6 µM FeSO_4_•7H_2_O, 1 µM CuSO_4_•5H_2_O, 130 nM CoCl_2_•6H_2_O, 24 nM (NH_4_)_6_Mo_7_O_24_•4H_2_O, and 1 µM ZnSO_4_•7H_2_O (basal) or without ZnSO_4_•7H_2_O (0-Zn).

0-Mg medium compose of 3 mM KNO_3_, 2 mM Ca(NO_3_)_2_•4H_2_O, 1.75 mM NaH_2_PO_4_•2H_2_O, 1.75 mM Na_2_HPO_4_•12H_2_O, 1.5 mM K_2_SO_4_, 1.5 mM K_2_SO_4_, 67 µM Na_2_-EDTA•2H_2_O, 30 µM H_3_BO_3_, 10.3 µM MnSO_4_•5H_2_O, 8.6 µM FeSO_4_•7H_2_O, 1 µM CuSO_4_•5H_2_O, 130 nM CoCl_2_•6H_2_O, 24 nM (NH_4_)_6_Mo_7_O_24_•4H_2_O, and 1 µM ZnSO_4_•7H_2_O.

0.5-Ca medium compose of 7 mM KNO_3_, 1.75 mM NaH_2_PO_4_•2H_2_O, 1.75 mM Na_2_HPO_4_•12H_2_O, 1.5 mM MgSO_4_•7H_2_O, 67 µM Na_2_-EDTA•2H_2_O, 30 µM H_3_BO_3_, 10.3 µM MnSO_4_•5H_2_O, 8.6 µM FeSO_4_•7H_2_O, 1 µM CuSO_4_•5H_2_O, 130 nM CoCl_2_•6H_2_O, 24 nM (NH_4_)_6_Mo_7_O_24_•4H_2_O, 1 µM ZnSO_4_•7H_2_O, and 0.5 mM CaCl_2_.

### 2.3. Determination of Elemental Contents

Five-day-old shoots were harvested from Col-0 grown on basal, 0-Zn, 0-Mg, and 0.5-Ca media. Roots were harvested and dried at 60 °C for two days. Dried samples weighing more than 10 mg were extracted with ultrapure HNO_3_ using a microwave digestion system (START D; Milestone General, Kawasaki, Japan). Elemental contents in the digests were determined by inductive coupled plasma mass spectroscopy (Agilent 7500cx; Agilent Technologies, Santa Clara, CA, USA). Measurements were performed with three independent biological replicates. 

### 2.4. iTRAQ-OFFGEL Fractionation Analysis

Each 20 µL of protein samples from protoplasts (2.5 mg mL^−1^) was reduced by tris-(2-carboxyethyl) phosphine at 60 °C for 60 min and then alkalized by methyl methanethiosulfonate at room temperature for 10 min. Samples were digested using 10mL of trypsin (1 mg mL^−1^) at 37 °C for 16 h. The digested peptides from protoplasts grown on basal, 0-Zn, 0-Mg, and 0.5-Ca media were labeled with iTRAQ-114, -115, -116, and -117 reagents, respectively, at room temperature for 60 min. The mixed peptide mixture was desalted on Sep-Pak C18 cartridges (Waters, Milford, MA, USA) followed by lyophilization to concentrate the peptides and then subjected to OFFGEL fractionation. The desalted peptides were mixed with the supplied OFFGEL buffer to obtain a 3.60 mL sample solution, which was then subjected to isoelectric focusing using immobilized pH gradient strips in the liquid phase [22]. The peptides were separated into 24 fractions with a 3100 OFFGEL fractionator (Agilent Technologies) using a 24 cm IPG gel, pH 3-10 (GE Healthcare, Little Chalfont, UK) at 4500 V for 50,000 Vh at 50 µA, according to the manufacturer’s instructions. The fractionated peptides were automatically purified using C-TIP (AMR, Tokyo, Japan); the details are described in [9].

### 2.5. LC-MS/MS Analysis

LC-MS/MS analysis was performed using an LTQ Orbitrap XL-HTC-PAL-Paradigm MS4 system. The iTRAQ labeled peptides were loaded onto the column (75-µm internal diameter, 15 cm, l-Column; CERI) using a Paradigm MS4 HPLC pump (Michrom BioResources, Auburn, CA, USA) and an HTC-PAL autosampler (CTC Analytics, Zwingen, Switzerland). Buffers were 0.1% (*v*/*v*) acetic acid and 2% (*v*/*v*) acetonitrile in water (A) and 0.1% (*v*/*v*) acetic acid and 90% (*v*/*v*) acetonitrile in water (B). A linear gradient from 5% to 45% B for 70 min was applied, and peptides eluted from the column were introduced directly into an LTQ Orbitrap XL mass spectrometer (Thermo Scientific, Waltham, MA, USA) at a flow rate of 200 nL min^−1^ and a spray voltage of 2.0 kV. The range of MS scan was *m*/*z* 450 to 1500 and the top three peaks were subjected to MS/MS analysis. The obtained spectra were compared against data in The Arabidopsis Information Resource (TAIR10; http://www.arabidopsis.org/) using the MASCOT server (version 2.4, Matrix Science, London, UK) and Proteome Discoverer (version 1.3, Thermo Scientific) with the following search parameters: threshold cut-off at 0.05 in the ion-score cut-off; protein identification cut-off set to two assigned spectra per predicted protein; peptide tolerance at 10 ppm; MS/MS tolerance at ±0.2 Da; peptide charge of 2+ or 3+; trypsin as the enzyme and allowing up to one missed cleavage; iTRAQ label and methyl methanethiosulfonate on cysteines as a fixed modification; and oxidation on methionine as a variable modification. iTRAQ data for three biological replicates were analyzed by MASCOT, and only the data with FDR < 1% were used for subsequent analyses. Only proteins that were identified in all three independent experiments were considered. The *P*-values were calculated by assuming that measurements approximated to a normal distribution.

### 2.6. Quantitative Real-Time RT-PCR Analysis

Col-0 roots grown for five days or protoplasts (~2.0 × 10^5^) from pGL2-GFPer and pMGP-GFPer were used to investigate the mRNA expressions of 14-3-3 protein family (*i.e.*, phi, chi, lambda, kappa, and epsilon). Total RNA was isolated from roots or protoplasts using an RNeasy Plant Mini Kit (Qiagen, Hilden, Germany). RNA was reverse-transcribed into cDNA using ReverTra Ace (Toyobo, Osaka, Japan) according to the manufacturer’s recommendations. Real-time PCR was performed using LightCycler^®^480 (Roche, Basel, Switzerland) with QuantiTect^®^ SYBR^®^Green PCR (Qiagen) in a total volume of 10 µL, including 12.5 or 6.9 ng of cDNA for roots or protoplasts, respectively. Gene-specific primers were described in Supplemental Table S3. The samples were normalized first to actin, and then the relative target gene expression was determined by performing comparative ∆∆C_T_. Three independent technical replicates using two independent sampling were performed for each gene. Statistical analysis was conducted using Student’s *t*-test.

## 3. Results and Discussion

### 3.1. Responsive Proteins in the Epidermis or Pericycle, Endodermis, and Cortex to Zn, Mg, and Ca Deficiency

**Figure 1:**
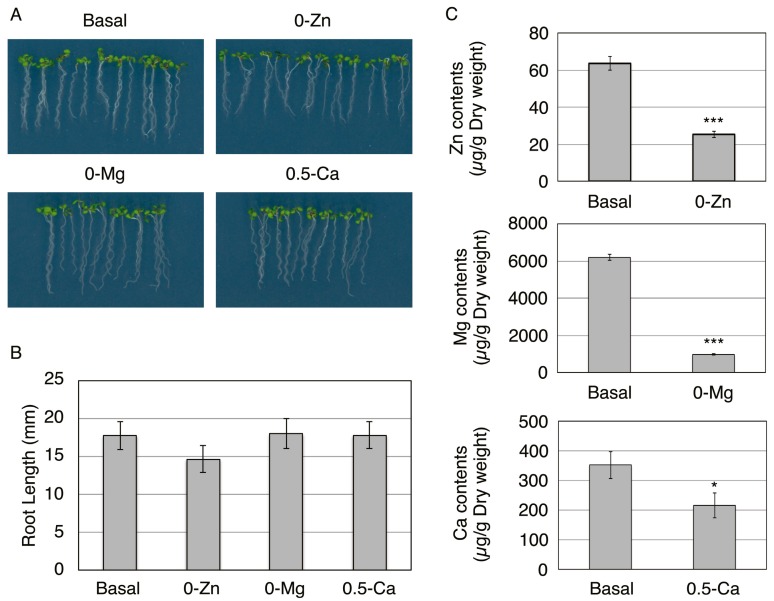
Effect of Zn, Mg, and Ca deficiency on plant growth. (**A**) Growth of Col-0 seedlings on basal, without Zn (0-Zn), without Mg (0-Mg), or medium containing 0.5 mM Ca (0.5-Ca), which is 25% Ca concentration in basal medium, for five days. (**B**) Root length of plants grown on each medium was measured. Data are shown as mean ± SD of four independent experiments. Ten seedlings were grown in each experiment at different times. (**C**) The concentrations of Zn, Mg, and Ca in roots grown on basal or 0-Zn, 0-Mg, or 0.5-Ca medium for five days were measured. Data are means ± SD of three independent experiments. * *P* < 0.05, *** *P* < 0.001 significantly different from the data on basal medium.

**Figure A1:**
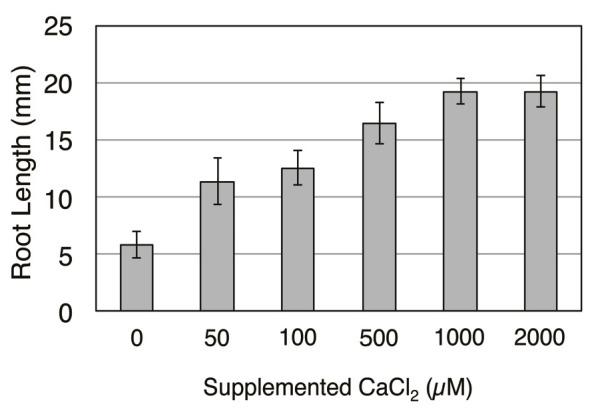
Phenotypes of Col-0 grown on a medium supplemented with or without Ca. Col-0 seedlings were grown on a medium supplemented with/without 0.05, 0.1, 0.5, 1.0, 2.0 mM CaCl_2_ for five days.

To identify the proteins responsive to essential elemental deficiency in each specific cell type, *Arabidopsis thaliana* Col-0 plants were grown on basal MGRL medium (basal), without Zn (0-Zn), without Mg (0-Mg), and with 25% Ca concentration in basal medium, for five days. These three deficient concentrations were determined based on the phenotypes of plants grown on media deficient in these three elements. Root growth on 0-Zn and 0-Mg media was slightly reduced or comparable to that observed on basal medium, but plant contents of Zn and Mg were significantly reduced (Figure 1). However, root growth was strongly inhibited when Col-0 plants were grown on medium without Ca (Figure A1). Therefore, we examined the growth inhibition at various Ca concentrations (*i.e.*, 0, 0.05, 0.1, 0.5, 1.0, and 2.0 mM). We chose these concentrations because the basal medium contains 2.0 mM Ca. Col-0 root growth was severely inhibited on 0, 0.05, and 0.1 mM Ca media and slightly inhibited on a 0.5 mM Ca medium (25% Ca concentration in basal medium), but it was not affected on 1.0 or 2.0 mM Ca media (Figure A1). Therefore, we examined the Ca concentration in Col-0 roots grown on a 0.5 mM Ca medium, and the Ca concentration was found to be significantly decreased (Figure 1C). Therefore, we chose a medium containing 0.5 mM Ca (0.5-Ca) as the Ca-deficient medium for this study. Next, we performed FACS sorting using roots grown in these three deficient conditions, as well as in basal conditions. 

**Figure 2:**
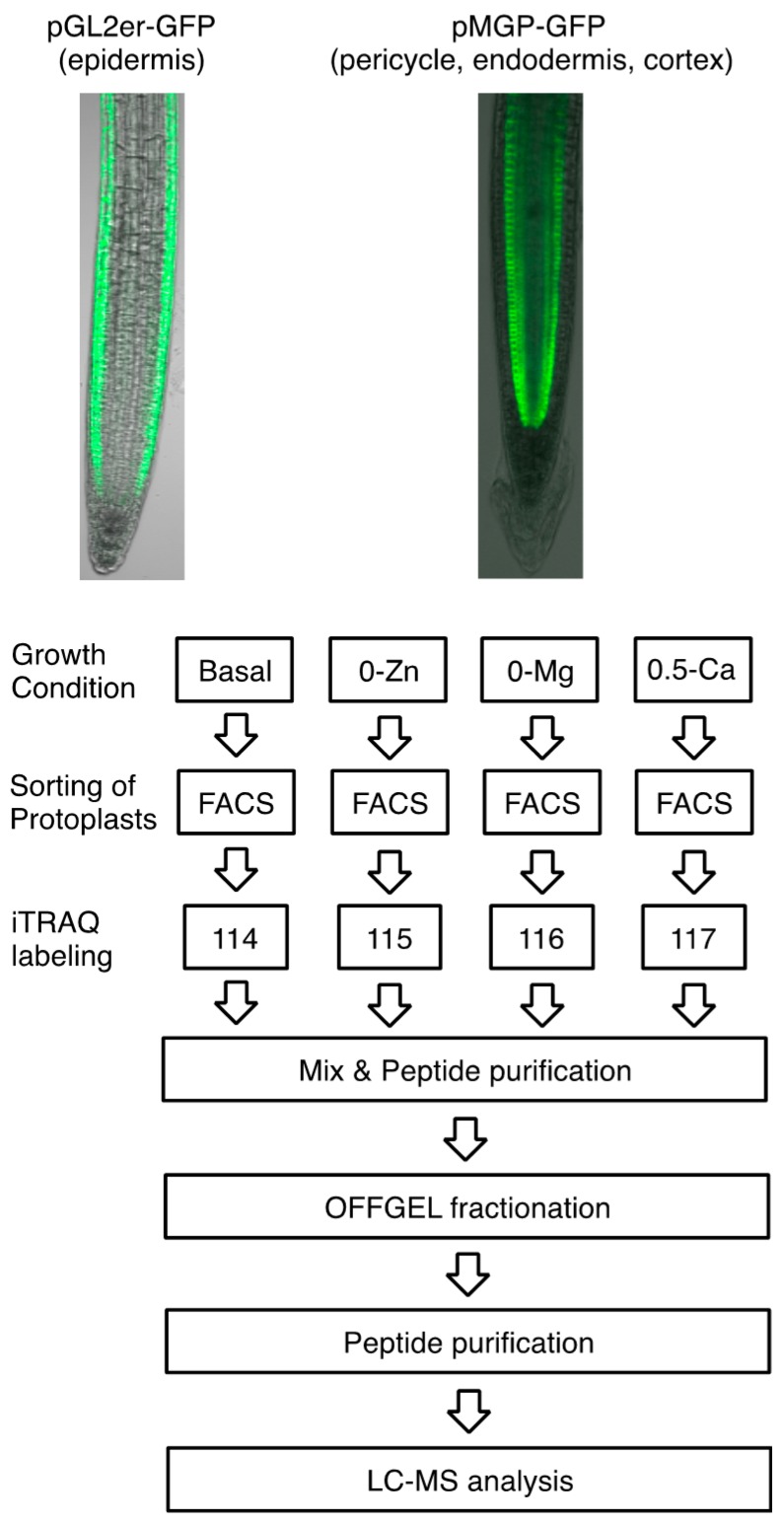
Experimental scheme used in this study. GFP-positive protoplasts were collected using FACS from five-day-old roots of pGL2-GFPer and pMGP-GFPer *Arabidopsis* transgenic lines grown on basal, without Zn (0-Zn), without Mg (0-Mg), or medium containing 0.5 mM Ca (0.5-Ca), which is 25% Ca concentration in basal medium, and used for iTRAQ-OFFGEL analysis.

We used pGL2-GFPer and pMGP-GFPer *Arabidopsis* transgenic plants as cell-type markers for epidermal and inner cells (*i.e.*, pericycle, endodermis, and cortex), respectively. The experimental scheme is summarized in Figure 2. Plants were grown on basal, 0-Zn, 0-Mg, and 0.5-Ca media for five days, and then protoplasts from each root sample were sorted by FACS, as described in our previous report [9]. The sorted GFP-expressing protoplasts were dissolved in iTRAQ buffer. Each 50-µg protein sample was subjected to iTRAQ labeling (114-, 115-, 116-, or 117-iTRAQ reagent), and labeled peptides were separated into 24 fractions by OFFGEL electrophoresis following peptide purification. The 24 fractionated peptide samples were analyzed by LC-MS following a subsequent round of peptide purification. To increase accuracy, this experiment was performed in triplicate for each cell type. 

**Figure 3:**
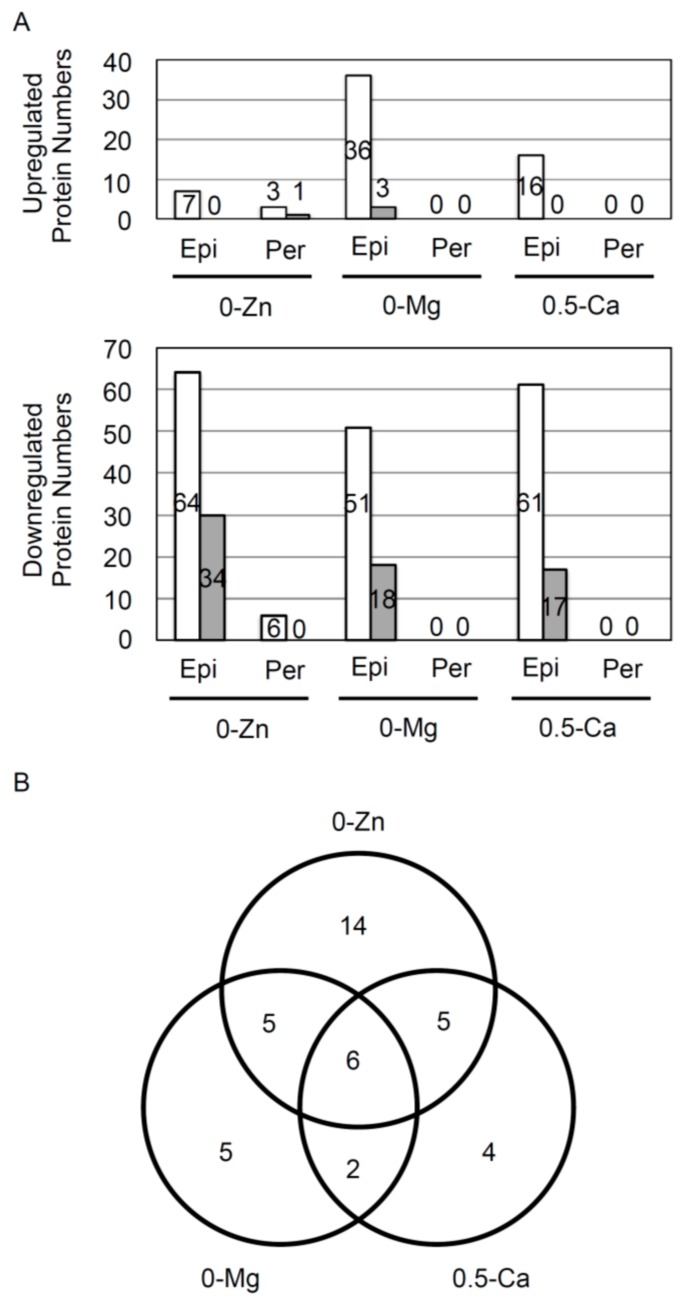
Summary of identified proteins.(**A**) The number of proteins whose expression increased by more than 1.5-fold or decreased by less than 0.667-fold are summarized in the different column chart. Upper and lower graphs show the number of proteins increased by more than 1.5-fold or proteins decreased less than 0.667-fold, respectively. **White** bars show proteins increased more than 1.5-fold and proteins decreased less than 0.667-fold, respectively. **Gray** bars show proteins increased more than 2.0-fold and proteins decreased less than 0.5-fold, respectively. Numbers inserted in the different column chart indicate the number of proteins identified. (**B**) Proteins decreased by more than 0.5-fold in response to Zn, Mg, and CPa deficiency in epidermal cells were compared.

Here, we identified and quantified a total of 1039 proteins from epidermal protoplasts and 737 proteins from inner cell protoplasts that were common in all three biological replicates with less than 1% FDR (Supplemental Tables S1 and S2). The number of strongly upregulated or downregulated proteins in each element-deficient condition are summarized in Figure 3A. Seven, 36, and 16 proteins increased more than 1.5-fold in the epidermal cells of plants grown on 0-Zn, 0-Mg, and 0.5-Ca, respectively. Sixty-four, 51, and 61 proteins decreased less than 0.667-fold in the epidermal cells of plants grown on 0-Zn, 0-Mg, and 0.5-Ca, respectively. However, only three and six proteins that were increased and decreased, respectively, were identified in the inner cells of plants grown on 0-Zn, and surprisingly, no proteins were observed to increase or decrease in plants grown on 0-Mg or 0.5-Ca (Figure 3A). Thus, the expression of many proteins was found to be decreased in the epidermis by mineral deficiencies, although protein expression remained almost unchanged in pericycle, endodermis, and cortex cells. These results indicate that the outermost cells may be most strongly affected by mineral deficiency. The casparian strips, which are split between the epidermal and endodermal cells in plant roots, have dual roles [23,24]. One important role of the casparian strip is the selective uptake of necessary or unnecessary materials from the soil. It also helps to prevent the diffusion of necessary materials, such as minerals, from central cells. Mutants of enhanced suberin1 (ESB1), which plays a role in the correct formation of casparian strips, show ectopic suberization [25]. In *esb1* mutant, the transport of Ca, Zn, and Mn to shoots is reduced [26]. Therefore, to sustain basic cellular functions, such as protein synthesis and metabolic pathways, in inner cell lines, consistent mineral concentrations must be maintained even when plants are suffering from mineral deficiency.

### 3.2. Overview of Proteins Strongly Downregulated by Zn, Mg, or Ca Deficiency in Epidermal Cells

We focused on the 41 proteins that were downregulated less than 0.5-fold in epidermal cells in response to mineral deficiencies (Figure 3B), although few proteins were upregulated more than 2.0-fold (Table 1). Fourteen, five, and four proteins were uniquely decreased by 0-Zn, 0-Mg, and 0.5-Ca, respectively. Six proteins were decreased in all three mineral-deficient conditions. However, no functional relationships among these six could be determined, except for the interaction between GF14 (14-3-3) protein and copper/zinc superoxide dismutase (SOD) in animal cells. It has been reported that 14-3-3 protein and SOD1 interact with tar DNA binding protein of 43 kDa (TDP-43) and modulate the stability of low molecular weight neurofilament mRNA [27]. Moreover, 14-3-3 protein phi chain (AT1G35160), GroES-like zinc-binding alcohol dehydrogenase family protein (AT3G15090), DEA(D/H)-box RNA helicase family protein (AT4G16630), and unknown protein (AT5G65810) were downregulated in response to mineral deficiencies in epidermal cells but not in inner cells (Table 2 and Supplemental Table S2). Another 37 proteins, which were decreased less than 0.5-fold in epidermal cells, were not found to be up- or downregulated in inner cells. Of the four proteins, we further examined the 14-3-3 protein, because 14-3-3 expression has been reported to be affected at the mRNA and protein levels by deficiencies of minerals such as potassium, phosphorous, and iron [28,29]. 

**Table 1:** List of proteins upregulated by more than 2.0-fold in epidermal cells.

AGI Code	Protein	0-Zn/basal (115/114)	0-Mg/basal (116/114)	0.5-Ca/basal (117/114)
AT1G48920	nucleolin like 1	1.63 ± 0.24	**2.03 ± 0.28**	1.71 ± 0.14
AT4G21960	Peroxidase superfamily protein	1.12 ± 0.18	**2.29 ± 0.47**	1.73 ± 0.37
AT5G62190	DEAD box RNA helicase (PRH75)	1.79 ± 0.27	**2.23 ± 0.38**	1.81 ± 0.16

Proteins in epidermal cells from roots grown on basal, without Zn (0-Zn), without Mg (0-Mg), or on a medium containing 0.5 mM Ca (0.5-Ca), which is 25% Ca concentration in basal medium, were labeled with iTRAQ-114, -115, -116, or -117 reagents, respectively. Proteins that were upregulated by more than 2.0-fold in comparison of 0-Mg treatment with the basal treatment were ordered based on AGI code. Data are means ± SD of three biological replicates. Mean values more than 2.0-fold are shown in bold font.

**Table 2:** List of proteins downregulated by less than 0.5-fold in epidermal cells.

AGI Code	Protein	0-Zn/basal (115/114)	0-Mg/basal (116/114)	0.5-Ca/basal (117/114)
AT1G01820	peroxin 11c	**0.40 ± 0.57**	0.68 ± 0.65	**0.38 ± 0.54**
AT1G07810	ER-type Ca^2+^-ATPase 1	**0.43 ± 0.74**	**0.45 ± 0.77**	0.58 ± 1.01
AT1G08830	copper/zinc superoxide dismutase 1	**0.46 ± 0.11**	0.90 ± 0.23	1.17 ± 0.23
AT1G12240	Glycosyl hydrolases family 32 protein	**0.43 ± 0.61**	**0.30 ± 0.42**	0.57 ± 0.81
AT1G14000	VH1-interacting kinase	0.53 ± 0.47	**0.41 ± 0.71**	N.D.
AT1G33490	unknown protein	0.62 ± 0.87	0.59 ± 0.84	**0.42 ± 0.60**
AT1G35160	GF14 protein phi chain	**0.33 ± 0.45**	**0.36 ± 0.39**	**0.49 ± 0.69**
AT1G35580	cytosolic invertase 1	**0.36 ± 0.51**	0.53 ± 0.75	**0.49 ± 0.69**
AT1G47420	succinate dehydrogenase 5	0.50 ± 0.71	**0.47 ± 0.66**	0.51 ± 0.72
AT1G49670	ARP protein (REF)	**0.37 ± 0.52**	0.63 ± 0.89	**0.23 ± 0.32**
AT1G71410	ARM repeat superfamily protein	**0.39 ± 0.67**	**0.38 ± 0.65**	**0.49 ± 0.85**
AT1G75330	ornithine carbamoyltransferase	**0.50 ± 0.70**	0.61 ± 0.86	0.59 ± 0.83
AT1G76550	Phosphofructokinase family protein	**0.49 ± 0.70**	**0.49 ± 0.70**	**0.46 ± 0.65**
AT1G77590	long chain acyl-CoA synthetase 9	**0.41 ± 0.58**	N.D.	N.D.
AT1G78800	UDP-Glycosyltransferase superfamily protein	0.82 ± 0.74	**0.34 ± 0.58**	1.00 ± 0.89
AT1G80600	HOPW1-1-interacting 1	**0.36 ± 0.62**	0.83 ± 0.72	0.69 ± 0.64
AT2G04305	Magnesium transporter CorA-like family protein	N.D.	**0.32 ± 0.46**	N.D.
AT2G17130	isocitrate dehydrogenase subunit 2	**0.47 ± 0.41**	**0.44 ± 0.63**	0.63 ± 0.89
AT2G28190	copper/zinc superoxide dismutase 2	N.D.	**0.40 ± 0.69**	**0.35 ± 0.60**
AT2G28520	vacuolar proton ATPase A1	**0.47 ± 0.66**	**0.44 ± 0.63**	0.50 ± 0.71
AT2G42260	uv-b-insensitive 4	**0.45 ± 0.64**	0.54 ± 0.76	0.56 ± 0.79
AT2G42500	protein phosphatase 2A-3	**0.41 ± 0.58**	**0.42 ± 0.60**	0.63 ± 0.89
AT2G42810	protein phosphatase 5.2	**0.24 ± 0.42**	0.56 ± 0.97	0.69 ± 1.19
AT2G45540	WD-40 repeat family protein / beige-related	**0.42 ± 0.59**	0.59 ± 0.83	0.60 ± 0.54
AT3G05280	Integral membrane Yip1 family protein	0.65 ± 0.23	0.82 ± 0.08	**0.40 ± 0.57**
AT3G13235	ubiquitin family protein	**0.33 ± 0.57**	0.92 ± 0.95	0.82 ± 0.77
AT3G15090	GroES-like zinc-binding alcohol dehydrogenase family protein	**0.46 ± 0.65**	0.66 ± 0.93	0.54 ± 0.76
AT3G15980	Coatomer; beta’ subunit	**0.42 ± 0.60**	1.17 ± 0.21	0.96 ± 0.00
AT3G23180	HR-like lesion-inducing protein-related	**0.42 ± 0.59**	0.54 ± 0.76	**0.47 ± 0.67**
AT3G28740	Cytochrome P450 superfamily protein	0.79 ± 0.79	**0.45 ± 0.42**	0.61 ± 0.57
AT3G44340	clone eighty-four	**0.36 ± 0.50**	**0.40 ± 0.57**	**0.33 ± 0.47**
AT3G48560	chlorsulfuron/imidazolinone resistant 1	**0.41 ± 0.71**	0.61 ± 1.05	0.54 ± 0.94
AT3G50590	Transducin/WD40 repeat-like superfamily protein	**0.40 ± 0.57**	0.51 ± 0.73	**0.38 ± 0.54**
AT3G52140	tetratricopeptide repeat (TPR)-containing protein	**0.17 ± 0.29**	0.71 ± 0.64	0.67 ± 0.63
AT4G05530	indole-3-butyric acid response 1	0.78 ± 0.75	0.75 ± 0.72	**0.46 ± 0.80**
AT4G14950	SNARE associated Golgi protein family	0.65 ± 0.57	0.51 ± 0.44	**0.37 ± 0.63**
AT4G16630	DEA(D/H)-box RNA helicase family protein	**0.38 ± 0.65**	**0.27 ± 0.47**	**0.27 ± 0.47**
AT5G14600	*S*-adenosyl-l-methionine-dependent methyltransferases superfamily protein	**0.40 ± 0.56**	0.84 ± 0.20	N.D.
AT5G54370	Late embryogenesis abundant (LEA) protein-related	**0.38 ± 0.66**	**0.36 ± 0.62**	**0.31 ± 0.54**
AT5G54640	Histone superfamily protein	**0.47 ± 0.66**	0.62 ± 0.88	0.60 ± 0.85
AT5G65810	unknown protein	0.60 ± 1.04	**0.37 ± 0.65**	**0.27 ± 0.48**

Proteins in epidermal cells from roots grown on basal, without Zn (0-Zn), without Mg (0-Mg), or on a medium containing 0.5 mM Ca (0.5-Ca), which is 25% Ca concentration in basal medium, were labeled with iTRAQ-114, -115, -116, or -117 reagents, respectively. Proteins that were downregulated by less than 0.5-fold in the comparison of any treatment with the basal treatment were ordered based on AGI code. Data are means ± SD of three biological replicates. Mean values less than 0.5-fold are shown in bold font. N.D. indicates that a peptide and/or iTRAQ reporter ions were not detected.

### 3.3. Transcriptional Response of Members of the 14-3-3 Protein Family in Epidermal or Pericycle, Endodermis, and Cortex Cells to Zn, Mg, or Ca Deficiency

**Figure 4:**
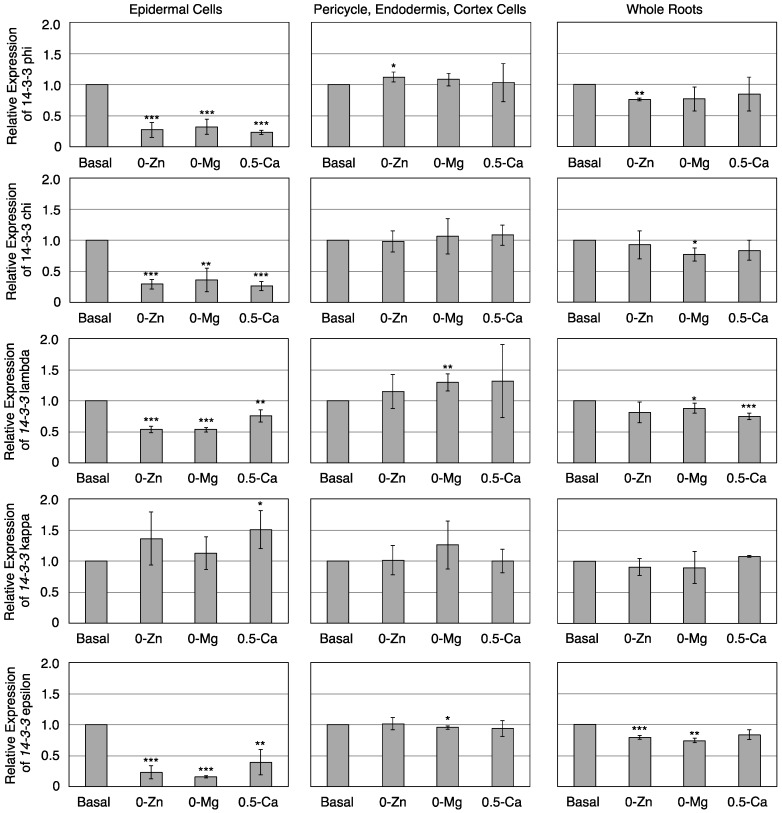
Real-time PCR analysis of *14-3-3s* using epidermal or pericycle, endodermis, and cortex cells and whole roots grown on basal, 0-Zn, 0-Mg, or 0.5-Ca medium. Relative transcript levels of 14-3-3 phi, chi, lambda, kappa, and epsilon from epidermal or inner cells (*i.e.*, epicycle, endodermis, and cortex) and whole roots grown on basal, without Zn (0-Zn), without Mg (0-Mg), or medium containing 0.5 mM Ca, which is 25% Ca concentration in basal medium, (0.5-Ca) medium. Data are shown as mean ± SD of three independent experiments. Expression on basal medium was adjusted to 1, as a relative unit. * *P* < 0.05, ** *P* < 0.01, *** *P* < 0.001 significantly different from the data on basal medium.

The 13 isoforms of the 14-3-3 protein family are classified into two groups: the non-epsilon group and the epsilon-like group [30,31]; each isoform shows a different capability to interact with client proteins [32]. Among the 13 isoforms, we examined 14-3-3 phi, chi, lambda, and kappa transcript levels in the non-epsilon group and 14-3-3 epsilon transcript levels in the epsilon-like group to Zn, Mg ,or Ca deficiency in epidermal or inner cells. The expression of 14-3-3 phi transcripts was significantly downregulated in response to Zn, Mg, or Ca deficiency in epidermal cells but not in inner cells (Figure 4). These results were completely consistent with the proteomic data. The expression of 14-3-3 chi, lambda, and epsilon transcripts was also significantly downregulated in epidermal cells, although the expression of 14-3-3 kappa transcripts was not. These results indicate that response to mineral deficiency is not correlated with the classification of the 14-3-3 protein family. In addition, these results suggest that not only protein expression levels but also mRNA expression levels are affected by mineral deficiency in epidermal but not in inner cells. Next, we examined the transcription levels corresponding to proteins in whole roots (Figure 4). Some 14-3-3 transcription levels were significantly downregulated by mineral deficiency, but less than in the epidermis. These unresponsive transcripts in other cell lines may offset the decrease in 14-3-3 protein transcripts in epidermal cells. These data also indicate the usefulness of the FACS-iTRAQ-OFFGEL method for identifying responsive proteins in each specific cell type.

The 14-3-3 protein has been shown to interact with plasma membrane H^+^-ATPase (AHA) or potassium channel. 14-3-3 proteins play a role in the activation of AHA via binding phosphorylation of the penultimate residue of AHA at the carboxy terminus [33,34]. 14-3-3 proteins have been shown to activate the potassium channel KAT1 on the plasma membrane for potassium uptake by heterologous protein expression analysis in *Xenopus* oocytes [35]. Furthermore, 14-3-3 proteins interact with the tonoplast potassium channel, TPK1, and strongly activate potassium transport activity [36,37]. Recently, it has been suggested that 14-3-3 proteins bind to mineral transporters including Zn, Mg, and Ca transporters [38,39,40,41]. Therefore, 14-3-3 proteins may play a role in nutrient accumulation. Indeed, it has been reported that 14-3-3 omicron plays a pivotal role in Fe accumulation by contribution to fer-like Fe deficiency-induced transcription factor (FIT) induction under Fe-deficient conditions [42]. Transcripts of iron-regulated transporter 1 (IRT1), ferric reduction oxidase 2 (FRO2), and AHA2, which are regulated by FIT, did not respond to Fe deficiency in 14-3-3 omicron mutant and failed to accumulate Fe. Moreover, 14-3-3 mu transcripts are downregulated by phosphorus deficiency and upregulated by replenishment with a sufficient concentration of phosphorus [43]. Therefore, downregulation of 14-3-3 expression under deficiency of several minerals can lead to suppression of mineral transport activities, and subsequent decreased mineral accumulation may inhibit plant growth. Furthermore, interactions between 14-3-3 proteins and target proteins might be disrupted at lower concentrations of Mg or Ca [44,45]. 

## 4. Conclusions

The FACS technique has been applied to investigation of the transcriptome of specific cell types [46] and to profiling the transcriptomic response to abiotic stimuli such as nitrate influx or iron deficiency [47,48]. Recently, transcriptomics using the FACS technique has been also applied to study of shoot apical meristem and leaves [49,50]. Moreover, this technique has been used in metabolomics to quantify auxin distributions [51] or glycosylated flavonols [52] and for proteomics analyses [8,9] in *Arabidopsis* roots. However, the FACS technique may have disadvantages, because the transcriptome, metabolome, and proteome may be altered during the protoplasting process. Alternative techniques for cell-type specific transcriptomics, including laser capture microdissection (LCM), isolation of nuclei tagged in specific cell types (INTACT), and immunopurification of ribosome-associated mRNA, have been developed [52]. Of these techniques, proteomics has been performed using LCM [53,54,55]. Nevertheless, specific cell-type proteomic techniques have not yet matured, and the advantages and disadvantages are still being investigated. In this study, we have performed first quantitative proteomics using particular plant cells collected by FACS. This technique will contribute to a better understanding of several cellular mechanisms in each particular cell types. However, to fully elucidate the changes in global proteome profiles in particular plant cells in response to various environmental stimuli, further technical developments and combinational analyses using specific cell-type collection methods are required.

## Supplementary Materials

The following are available online at www.mdpi.com/2227-7382/4/1/1/s1. Table S1: List of all proteins identified by FACS-iTRAQ-OFFGEL analysis in epidermis of roots grown on basal, 0-Zn, 0-Mg, or 0.5-Ca medium. Table S2: List of all proteins identified by FACS-iTRAQ-OFFGEL analysis in pericycle, endodermis, and cortex from roots grown on basal, 0-Zn, 0-Mg, or 0.5-Ca medium. Table S3: Primer sequences for qRT-PCR.
